# Observable phenomena that reveal medical students' clinical reasoning ability during expert assessment of their history taking: a qualitative study

**DOI:** 10.1186/s12909-017-0983-3

**Published:** 2017-08-29

**Authors:** Catharina M. Haring, Bernadette M. Cools, Petra J. M. van Gurp, Jos W. M. van der Meer, Cornelis T. Postma

**Affiliations:** Department of Internal Medicine (463), Radboud University Medical Center, PO Box 9101, 6500 HB Nijmegen, the Netherlands

**Keywords:** Clinical reasoning, History taking, Assessment, Undergraduate medical education

## Abstract

**Background:**

During their clerkships, medical students are meant to expand their clinical reasoning skills during their patient encounters. Observation of these encounters could reveal important information on the students’ clinical reasoning abilities, especially during history taking.

**Methods:**

A grounded theory approach was used to analyze what expert physicians apply as indicators in their assessment of medical students’ diagnostic reasoning abilities during history taking. Twelve randomly selected clinical encounter recordings of students at the end of the internal medicine clerkships were observed by six expert assessors, who were prompted to formulate their assessment criteria in a think-aloud procedure. These formulations were then analyzed to identify the common denominators and leading principles.

**Results:**

The main indicators of clinical reasoning ability were abstracted from students’ observable acts during history taking in the encounter. These were: taking control, recognizing and responding to relevant information, specifying symptoms, asking specific questions that point to pathophysiological thinking, placing questions in a logical order, checking agreement with patients, summarizing and body language. In addition, patients’ acts and the course, result and efficiency of the conversation were identified as indicators of clinical reasoning, whereas context, using self as a reference, and emotion/feelings were identified by the clinicians as variables in their assessment of clinical reasoning.

**Conclusions:**

In observing and assessing clinical reasoning during history taking by medical students, general and specific phenomena to be used as indicators for this process could be identified. These phenomena can be traced back to theories on the development and the process of clinical reasoning.

## Background

Clinical reasoning is considered to be the foundation of patient care, and, as such, clinical reasoning is part of every endeavour clinicians make in clinical care, enabling them to take the best justified action in a specific context [8].

Theories on clinical reasoning all point out that this process starts with data gathering, which initially takes place during history taking [1]. The performance of history taking, therefore, could tell us a great deal about the clinicians’ skill to start diagnostic reasoning, which is the initial phase of clinical reasoning. If this step fails, it is hard to see how an accurate diagnosis might be reached. Physical examination, of course, could also provide additional information for defining a proper differential diagnosis, but, in most clinical encounters, this is a separate process that follows the initial history taking.

Clinical reasoning as a concept emphasizes the cognitive process involved rather than its endpoint, the diagnosis [15]. To reach a diagnosis, a connection must be made between existing knowledge and data obtained. If this connection is lacking, this lack will be reflected in how the process is carried out as, in essence, one may gather a substantial quantity of data and yet have no idea what they mean.

Monitoring the acquisition of clinical reasoning skills by medical students and other trainees in the clinical domain is essential. Clinicians are familiar with monitoring their trainees’ clinical reasoning in the workplace and are often readily able to identify those who are poor at it. The most important point here is that there are no unequivocal criteria for assessing students’ clinical reasoning during a clinical encounter and that such assessment, therefore, is often informal, implicit, not standardized and could be based on holistic or subjective judgments. What happens in practice is that, after the encounter and the write up, the teacher asks for a differential diagnosis and the elements that lead up to it [1]. In terms of patient care, this could be a careful way of acting, but from an educational point of view, it might not be accurate enough. What is actually assessed in this case is the endpoint of the thought process that results in a clinical decision or clinical judgment. Before that endpoint is reached, however, the process of diagnostic reasoning takes place, which includes the cognitive processes leading to the endpoint. If it is only the outcome that is discussed, these cognitive processes go unnoticed and uncorrected [15].

Providing adequate and targeted feedback enables further development. To be able to provide feedback, teachers must observe and assess what students are doing. Providing feedback on clinical reasoning and assessing it, therefore, are intertwined elements of the same process. If this is not properly observed, therefore, feedback might be inadequately targeted and fall short of its intended purpose [9]. Many clinical exercises in which students take a history are ideally suited to formally assess the initial part of the clinical reasoning process, so-called diagnostic reasoning [11]. Although the importance of this procedure is well recognized, the assessment of clinical reasoning in these encounters in clinical practice still appears to be a major struggle for clinicians [4, 5].

Identifying observable phenomena representing the cognitive processes of diagnostic reasoning would allow us to gauge students’ skills during the process of clinical reasoning. This would give us the tools on which to base feedback and assessment, with the purpose of improving the development of these skills in students. The question then arises how we can reliably assess what is actually shown by students as an expression of diagnostic reasoning during history taking. To be able to answer this question, we should first know what can actually be observed regarding clinical reasoning in the learners’ behaviour during history taking.

To assess competence in clinical reasoning, test situations should be as realistic as possible and should be based on sufficient numbers of clinical examples [17]. These two ingredients are abundantly present during clinical clerkships [1]. Most of the initial information needed for a proper differential diagnoses is acquired by careful and adequate history taking [7]. However, if diagnostic reasoning is to be observed, it must be deduced from students’ observable behaviour [10], which could lead to large interrater variability. In addition, a clinical setting cannot be standardized, like case presentations in an OSCE, whilst it is known that even small changes in context or content can affect clinical reasoning performance [2]. This phenomenon, referred to as ‘context or content specificity’, is often seen as a major obstacle to assessing medical students’ clinical reasoning as it precludes generalizability. On the other hand, it is exactly the ever-changing contexts in daily medical practice that make clinical reasoning such a real challenge, even more so as the object of assessment [3]. Therefore, we need to define the phenomena and criteria that reflect the process as an observable action.

We hypothesize that it is possible for experts to recognize and identify phenomena and patterns that indicate proper diagnostic reasoning and that, by identifying these, valid assessment criteria for diagnostic and clinical reasoning in clinical practice could be developed. A necessary prerequisite for the use of such criteria is that they should be in accordance with what are considered to be the generally accepted concepts and theories of clinical reasoning.

Hence, our main research question is: Which observable phenomena serving as indicators for clinical reasoning do experts identify by observing students during history taking?

## Methods

### Setting and sampling

The study was conducted in our university medical center. The present curriculum is a six- year competency-based curriculum with a Bachelor’s and a Master’s programme. Clinical reasoning skills are taught throughout the Bachelor’s programme. An internal medicine clerkship is part of the first year of the three-year Master’s programme, which involves training in clinical practice. The internal medicine outpatient clinic has two consulting rooms that have been prepared with recording systems; these are used by students on a daily basis to record their consultations for training purposes.

Principal lecturers (PLs) participated in this study. The Radboud university medical center appoints excellent medical teachers who, as principal lecturers, play a leading role in teaching and developing medical education. All are practicing clinical physicians. The acquirement of PL status allows them to dedicate more time to developing medical education in all its facets. The department of internal medicine has seven principal lecturers among its faculty. One of the principal lecturers (CP) was too closely involved with the design of the study to be included as a potential participant. All other PLs, three males and three females, were invited to participate on a voluntary basis, to which all agreed.

### Design

To be able to contribute theory formation about assessment of clinical reasoning, we chose a grounded theory methodology [16], which addresses knowledge gain through the construction of new theories that are grounded in a systematic analysis of the data.

### Data collection

The PLs were invited to participate in one-hour research sessions. These sessions took place in a quiet room with only the researcher and the PL present. The six PLs studied two different recordings of medical students’ history taking in a new patient encounter. These consultations by the students were recorded during the last week of their internal medicine clerkships. Prior to recording, patients were asked for their permission to use the recordings for educational purposes. Twelve recordings were randomly chosen from a larger number and were screened briefly to prevent inclusion of similar case presentations. The PLs were asked to watch the process and stop the recording when they thought they noticed the clear presence or absence of signs of overt clinical reasoning. At those points, the PLs were prompted to think aloud on what they observed and how they noticed that the cognitive process of clinical reasoning was active or lacking. Recordings could also be interrupted by the researcher when stimulation of the think-aloud process was considered necessary. The participants were not prompted to rate or judge students. During this sessions audio recordings of the think-aloud process were made for data analysis.

### Analysis

Data analysis was started during the data collection process so that emerging themes could be explored in more depth with subsequent participants. The audio recordings were transcribed to written text for further analysis. Initial themes were explored (open coding), alternated with exploration of interrelationship amongst these themes (axial coding) to identify patterns and main categories in the data and stimulate conceptual understanding [16]. This process was carried out by the principal researcher (CH). Renaming of themes and determination of relationships between the themes were done by the principal researcher in collaboration with the research team, leading to the development of a conceptual framework. After analysis of all twelve interviews, data saturation was discussed and approved by the research group.

### Validation

The emerging themes were presented and discussed with the participants to ensure that these resonated with their perceptions. Their vision was used to refine the conceptual framework.

## Results

### 1. Indicators for clinical reasoning

Whilst watching the recordings, the PLs saw general indicators for the process of clinical reasoning emerging at four different levels of the encounter. These were: I students’ acts; II patients’ acts; III the course of the conversation; and IV the result of the conversation and efficiency (Table 1). Different indicators could appear per level. All indicators emerged as themes in half to all of the interviews with the participants (Table 1).

**Table 1:** Indicators for clinical reasoning

	Number of times the indicator was mentioned by the participant (A-F)						Quotes
Indicator	A	B	C	D	E	F	
I Student s’ acts							
Taking the lead in the conversation	8	5	6	7	15	3	‘She allows the patient to talk for too long. It doesn’t become clear to me whether she does so on purpose, to organize everything directly in her head. I do not see that happen. It appears to me that she doesn’t have a clue what to do at this point and she’s waiting for the golden tip to arise.’ ‘She allows the patient tell the same story again. She doesn’t give any direction. That’s why I think she doesn’t know what to do with that information.’ ‘I think other students would probably allow her to go on talking, and then the story might end with a different subject, for example the adventures of her friend.’
Recognizing and responding to relevant information	8	3	9	10	5	10	‘Apparently now she’s making a choice; the health problem that occurred four, five weeks ago, that’s the problem she has to get started with. Here she reaches the point that she takes this particular problem from the whole pool of problems. So here clinical reasoning is going on.’ ‘Sometimes you think she gets it, but then she’s losing it. She doesn’t proceed to ask questions at a crucial moment, the moment when the patient signals something that makes the story special.’
Specifying symptoms	4	5	6	12	22	3	‘She knows that she should not be satisfied when a patient mentions “my stools aren’t normal”.’ ‘She knows she has to ask additional questions. Apparently, she doesn’t have the eagerness to continue questioning when it comes to shoulder pain. Apparently, she’s satisfied and she doesn’t realize this by asking more questions about that problem to show differential diagnostic reasoning.’ ‘He’s going off track. The patient was dizzy for one day at the start of her period. Well, I wouldn’t want to meet all women who have that once in a while at the start of their period. And now he’s asking about that in depth, while he should go on with her complaining of fatigue.’
Asking specific questions that point to pathophysiological thinking	10	6	14	3	3	14	‘She asked about stress. So she is thinking of a stomach ulcer, or reflux.’ ‘What I notice, is that she’s trying to find out whether there’s a bowel obstruction. At least she appears to be doing that with these questions.’ ‘He’s asking the regular questions that belong to the history of pain. He is trying to get a grip on it. Which is proper. But he’s not having a differential diagnosis at this moment.’ ‘She really doesn’t know what she’s talking about. She is simply completing her list.’
Putting questions in a logical order	3	3	2	2	14	2	‘Why she returns to the hands, while in fact we don’t know the context of the knee problem is totally unclear to me.’ ‘Now she’s clustering questions that belong together, questions that indicate heart failure.’
Checking with the patient	0	3	4	2	2	0	‘This is a question of which you would expect there’s something in it … You can’t accept only “no”, as this patient answered. I would ask her additional questions to be sure it’s really “no”. And to be sure that the patient understands what you mean by that question.’
Summarizing	1	1	6	1	0	1	‘The way she is summarizing, what she chooses to summarize, which topics she chooses and how she describes them, all this gives me an idea at what point she has arrived in her history taking. It shows what she thinks the problem with this patient may be.’
Body language	0	1	5	0	0	1	‘When I look at her now, I see a despondent look on her face, expressing: what should I do?’ ‘The look on her face that says, well, what should I be aiming at? What’s the problem I should be analyzing?’
II Patients’ acts							
Patient taking the lead	2	1	3	2	6	1	‘Look, now the patient is taking over. Now she leads the student back to her problems because the student isn’t doing it.’
III Course of the conversation							
Talking at cross-purposes	2	0	4	2	2	1	‘There’s a misunderstanding between the doctor and the patient. The patient is pointing at her skin and mentions pain in her leg, but the student is talking about her knee problem.’ ‘By the way, we don’t know now whether he understands it all, and they talk at the same level. He’s talking about the period before Christmas, and he thinks she describes the symptoms she had when she was first using milk products. But I’m not sure whether she is talking about that period.’
Repetition	5	1	0	0	0	4	‘With regard to clinical reasoning, he’s gained his information in a more efficient way than the female student. She needed a lot of time. She didn’t realize that she had already gained a lot of information. She allowed the patient to tell the story three times.’
IV Data gathered and efficiency							
Data gathered	0	2	0	2	5	2	‘He hasn’t gained enough specific information.’ ‘A couple of questions like palpitations, tremors, I’m still missing.’
Efficiency	12	1	3	0	11	3	‘But only now, after how much time? Eight minutes, she’s getting somewhere.’

Indicators for clinical reasoning pointed out by experts during assessment of medical students. The number of times an indicator was mentioned during the research session is displayed (participant A-F). Each indicator is illustrated by one or more quotes from the participants

#### Level I students’ acts

Most comments on indicators of clinical reasoning were linked to the students’ performance. In general, the students had to show that they were actually processing the information and were making choices during the conversation. The students were expected to show curiosity and play an active role in the conversation.

##### Taking the lead in the conversation

Clinical reasoning was assessed by the way students took the lead in the conversation. Students had to show that they could direct the conversation towards the information they needed to know. This did not mean that clinical reasoning was assessed negatively when students allowed patients to tell their own story, but if students failed to actively take the lead at a certain moment, they did not show that clinical reasoning was taking place.

##### Recognizing and responding to relevant information

Students had to show that they recognized relevant information by the way they responded to this information. If the expert assessors recognized an important clue in the patients’ story and the students also responded to this clue with interest, the observer perceived that clinical reasoning was taking place. Not responding to clues that were marked as important by the expert assessors was interpreted as the absence of clinical reasoning or as wrong clinical reasoning.

##### Specifying symptoms

When students had identified a leading health problem, the one that was worth pursuing, it had to be explored in depth. By showing eagerness and curiosity to specify symptoms that seemed important, students showed clinical reasoning according to the expert assessors. This included localization of the health problem and its evolution in time. Specifying symptoms could also be assessed negatively when their relevance was doubted by the expert assessors.

##### Asking specific questions pointing to pathophysiological thinking

Students could ask questions that were not direct responses to what the patients said, but that were evidence of hypothesizing about specific causes of a health problem. The assessors might have recognized this hypothesis by the specific questions asked. Instead of asking specific questions pointing to a specific diagnosis, some students appeared to be just reproducing lists of questions they had learned to use. If they did this too often, a negative assessment was given for clinical reasoning.

##### Putting questions in a logical order

Questions should be placed in a logical order. If the students failed to do so, the assessors did not recognize that the students were applying clinical reasoning.

##### Checking with patients

Based on comments of the expert assessors, students should show that they were checking with patients to be sure that their clinical reasoning was going in the right direction. The assumption here is that appropriate clinical reasoning is only possible when it is based on correct data. Principal lecturers expect their students to check constantly whether they are gathering the right information. Not only did they have to ensure that they understood patients, but they also had to verify that patients understood them so as to be able to make differential diagnoses based on the correct information.

##### Summarizing

Students are taught to summarize aloud when they have obtained a sizable quantity of information. Such summaries are used by the principal lecturers to judge clinical reasoning.

##### Body language

Body language was also an element to be observed in clinical reasoning. For example, the assessors recognized confusion on the students’ faces or in their postures.

#### Level II patients’ acts

Assessors could interpret patients’ acts as indicators for students’ clinical reasoning when the students’ clinical reasoning ability was reflected by the patients’ acts. When patients felt that students did not understand the problem, patients could show a tendency to take over the leading role. This was interpreted as a negative sign for clinical reasoning.

#### Level III course of the conversation

Students’ acts as well as patients’ acts influenced the course of the conversation. Some phenomena, or patterns, might appear that were caused by acts of either the students or the patients and that reflected the students’ clinical reasoning ability. When students and patients were talking at cross purposes, data gathering was believed to be insufficient. Repetition either induced by students or patients could occur during the conversation and was considered a negative indicator for clinical reasoning.

#### Level IV data gathered and efficiency

The quantity and quality of gathered data as well as the speed of data gathering (efficiency) were regarded as indicators of clinical reasoning being in progress. If students took too long to get to an essential point, their clinical reasoning ability was assessed negatively. This was also mentioned for the quantity and quality of data that had been gathered by the end of the session. If, according to the assessors, students failed to obtain a sufficient quantity of data to make a proper differential diagnosis, their clinical reasoning was assessed negatively.

### 2. Performance assessment of clinical reasoning

**Fig. 1:**
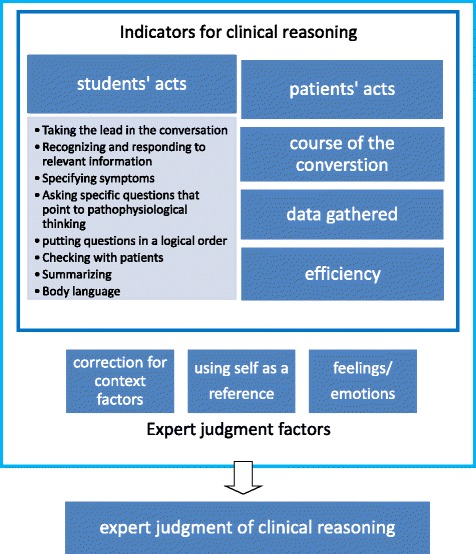
Conceptual model for expert judgment of clinical reasoning of medical students through observation of history taking

The PLs were not asked to rate or assess students actively. However, pointing out the presence or absence of indicators for clinical reasoning during assessment of clinical reasoning often automatically resulted in assessment of clinical reasoning. By using a grounded theory approach, information about the assessment of competence in clinical reasoning emerged, which led to a conceptual framework (Fig. 1). Correcting for context factors, using self as a reference and undefined feelings and emotions were the three themes that influenced the assessment of competence in clinical reasoning by the expert assessors, as will be explained below.

#### Correcting for context factors

When expert assessors felt the need to give an opinion about the quality of clinical reasoning that was presented to them, they often corrected their assessment based on context factors. Context factors that could be derived from this study were: patient factors, disease factors, student factors and other external factors. When context factors appeared to make clinical reasoning more difficult, for example when the student gets interrupted by external factors, or when the patient is verbose, it affects the judgement of the assessors.

#### Using self as a reference

The assessment of the experts appeared largely to be influenced by choices they thought they would make themselves at a certain point in history taking. By taking themselves as a frame of reference, assessors compared the student performance with how they perceived themselves to practice.

#### Emotions/feelings

Although many observable phenomena serving as indicators for assessing clinical reasoning could be distinguished, not all observable phenomena could be put into words by the expert assessors. Often observations then were expressed as emotions or feelings. Assessors for example expressed that they could feel that clinical reasoning was happening.

## Discussion

In this study, expert physicians – as expert raters of clinical reasoning – identified a set of indicators that capture basic elements of students’ diagnostic reasoning during a clinical encounter, in particular history taking. These indicators can be used to assess this process in a structured and reliable way in the setting of the presumptive diagnostic reasoning early in the clinical encounter.

Although diagnostic reasoning can be viewed from different epistemological angles [4, 5], in this context it is conceived as a cognitive process of drawing conclusions or making inferences from the facts or premises obtained early on in the process of clinical reasoning. By identifying the criteria by which this early stage of clinical reasoning can be observed and assessed, a tool can be developed specifically targeting this important point in medical students’ clinical training.

The more one studies clinical experts, as some authors observe, the more one marvels at the complex and multidimensional components of knowledge and skill they bring to bear on the problem and the amazing adaptability they must possess to achieve the goal of effective care [12]. Fundamentally, this can be defined as a complex cognitive process that involves formal and informal thinking strategies to gather and analyze patient information, evaluate the significance of the information and weigh alternative actions [15]. This is a dynamic process; information that is initially discarded may be retrieved later in the process. As experience builds up and pattern recognition becomes an integral part of the process, this allows for faster procedures. Novices may be more limited to relying on prototypical or analytic knowledge and information processing, having fewer exemplars or experiences to draw upon [14].

This process of data handling, searching for clues and significance and gathering and analyzing data obtained, is reflected in the students’ clinical diagnostic reasoning during history taking. This is the initial data gathering process. On the basis of these considerations, we could probably identify indicators of the process of clinical reasoning (or diagnostic reasoning, at this initial stage), regardless of the patients’ context or the problem they present. These indicators are the students’ observable actions to acquire meaningful information. The proper use or expression of these indicators could then enable us to standardize and thus generalize the way in which expert clinicians could judge medical students’ clinical reasoning in this respect. This could provide clinical teachers with more reliable and structured clinical assessment and feedback methods to monitor the performance of clinical reasoning in the initial stages of clinical training.

It has been observed that the role of inferences is prominent in performance assessment [10]. Inferences are conclusions that come at the end of a chain of reasoning. We show that inferences can be based on recognition of the prespecified objective and, therefore, on more reliable criteria. As a result, such criteria and patterns can be recognized by different experts observing different students in different contexts. Others have also suggested that these inferences can in fact be markers of expertise, which represent the assessors’ ability to tap into well-developed assessment schemes [18]. In our study, we have identified workable criteria to judge the process of information gathering and analysis that forms the basis of the medical students’ clinical – diagnostic – reasoning process. These criteria, as formulated by the clinical experts, are in line with the existing concepts of the process of clinical – diagnostic – reasoning.

It is no surprise that the students’ acts would play a major role in the observable indicators for clinical reasoning. When patients take the lead, however, the students’ acts are also thought to be a reflection of their lacking clinical reasoning ability. Our main research question involved a search for common observable indicators for clinical reasoning, but our research method, involving a think-aloud process, apparently also stimulated performance assessment, even though the participants were not actively stimulated to rate or assess the students. In line with current literature [10], we found that expert assessors constantly incorporate context factors into their assessment and use frames of reference. When it comes to clinical reasoning, however, it is notable that expert assessors mainly appear to consider themselves as a reference. The extent to which assessors are capable to correct their judgement for context factors and to apply their own frame of reference to the student’s performance might be the reflection of their expertise.

In social perception theory, assessors or raters can be seen as social perceivers who use motivated social assessments when evaluating performance and use pre-existing knowledge structures or schemes when assessing performance. These schemes are based on the expectation that assessors evaluate the students’ behaviour in a certain social position, in specific social situations or as persons [6]. Hoping to improve the assessment of clinical reasoning in practice, we feel that research should also focus on how these internal frames of reference or internal standards for assessing clinical performance develop. We need to improve our understanding of the underlying processes that influence the development of internal standards that are used for assessing performance. This knowledge could be helpful in training performance assessment and could serve as a basis for personalized feedback in the professional training of students and residents.

Ultimately, all medical students need to be assessed on whether they are evolving satisfactorily as medical professionals, an essential part of which is the development of appropriate clinical reasoning skills. One student failing to show clinical reasoning ability was strikingly described by one of our expert assessors as ‘someone who is engaged in social talk but not in professional exchange’. In this study, we could outline indicators that experts can use to distinguish clinical reasoning from ‘social talk’. This might be a very useful supplement for clinicians to assess and stimulate the improvement of their trainees’ clinical reasoning ability. Our next step is to asses validity and reliability of these indicators when they are used to assess students’clinical reasoning ability in clinical practice.

### Limitations

Though the number of participants was limited, all themes emerged from half to all of the participants, and no new themes emerged; data saturation, therefore, was considered complete. We are aware that cultural differences might have influenced the way in which assessment of clinical reasoning was addressed by expert assessors. The conceptual framework, therefore, could be enriched by repeating the study in other countries or other medical disciplines.

There were large variations in the number of times that themes appeared during different sessions. This could be due to variety amongst the participants as well as variety among the presented cases. We are well aware that assessment only by observing the students has limitations. The students’ explicit thought process during the clinical encounter was not an integral part of the assessment. Therefore, we do not advocate assessment of clinical reasoning from this perspective only. Obviously, observation also takes precious time. Different methods could complement each other. Direct observation, however, is not always part of daily routine as it should be and takes place only rarely during most clerkships [13].

The study was performed in Dutch but reported in the English language; some nuances, for example in quotes, might have been lost.

## Conclusions

Expert assessors use general indicators while assessing students’ clinical reasoning during history taking. As such, these indicators could satisfy the need for a common way of assessing clinical reasoning in a practical setting such as an outpatient clinic.

## Abbreviation

PL: Principal lecturer

## References

[1] Bowen JL (2006). Educational strategies to promote clinical diagnostic reasoning. N Engl J Med.

[2] Durning S, Artino AR, Pangaro L, van der Vleuten CP, Schuwirth L (2011). Context and clinical reasoning: understanding the perspective of the expert’s voice. Med Educ.

[3] Durning, S. J., Artino, A. R., Boulet, J. R., Dorrance, K., van der Vleuten, C., & Schuwirth, L. (2012). The impact of selected contextual factors on experts’ clinical reasoning performance (does context impact clinical reasoning performance in experts?). Adv Health Sci Educ Theory Pract*,* 17(1), 65-79. doi:10.1007/s10459-011-9294-3. 10.1007/s10459-011-9294-3 21505841

[4] Durning SJ, Ratcliffe T, Artino AR, van der Vleuten C, Beckman TJ, Holmboe E (2013). How is clinical reasoning developed, maintained, and objectively assessed? Views from expert internists and internal medicine interns. J Contin Educ Health Prof.

[5] Durning SJ, Artino AR, Schuwirth L, van der Vleuten C (2013). Clarifying assumptions to enhance our understanding and assessment of clinical reasoning. Acad Med.

[6] Govaerts MJ, Van de Wiel MW, Schuwirth LW, Van der Vleuten CP, Muijtjens AM. Workplace-based assessment: raters’ performance theories and constructs. Adv Health Sci Educ Theory Pract. 2012; doi:10.1007/s10459-012-9376-x. 10.1007/s10459-012-9376-x PMC3728456 22592323

[7] Hampton JR, Harrison MJ, Mitchell JR, Prichard JS, Seymour C (1975). Relative contributions of history-taking, physical examination, and laboratory investigation to diagnosis and management of medical outpatients. Br Med J.

[8] Higgs J, Jones M, Loftus S, Christensen N (2008). Clinical reasoning in the health professions.

[9] Howley LD, Wilson WG (2004). Direct observation of students during clerkship rotations: a multiyear descriptive study. Acad Med.

[10] Kogan JR, Conforti L, Bernabeo E, Iobst W, Holmboe E (2011). Opening the black box of clinical skills assessment via observation: a conceptual model. Med Educ.

[11] Norcini J, Burch V (2007). Workplace-based assessment as an educational tool: AMEE guide no. 31. Med Teach.

[12] Norman G (2005). Research in clinical reasoning: past history and current trends. Med Educ.

[13] Pulito AR, Donnelly MB, Plymale M, Mentzer RM (2006). What do faculty observe of medical students’ clinical performance?. TeachLearnMed.

[14] Schmidt HG, Rikers RM (2007). How expertise develops in medicine: knowledge encapsulation and illness script formation. Med Educ.

[15] Simmons B (2010). Clinical reasoning: concept analysis. J Adv Nurs.

[16] Strauss A, Corbin J (1990). Basics of qualitative research. Grounded theory Prodedures and techniques.

[17] van der Vleuten CP, Newble DI (1995). How can we test clinical reasoning?. Lancet.

[18] Yeates P, O'Neill P, Mann K (2011). Examining the box's contents. Med Educ.

